# Translational potential of a mouse *in vitro* bioassay in predicting gastrointestinal adverse drug reactions in Phase I clinical trials

**DOI:** 10.1111/nmo.12349

**Published:** 2014-05-11

**Authors:** C Keating, L Ewart, L Grundy, JP Valentin, D Grundy

**Affiliations:** * Department of Biomedical Sciences, University of SheffieldSheffield, UK; † Department of Safety Pharmacology, Global Safety Assessment, AstraZeneca R&D Alderley ParkMacclesfield, UK

**Keywords:** adverse drug reaction, bioassay, biomarker, gastrointestinal motility, safety pharmacology

## Abstract

**Background:**

Motility-related gastrointestinal (GI) adverse drug reactions (GADRs) such as diarrhea and constipation are a common and deleterious feature associated with drug development. Novel biomarkers of GI function are therefore required to aid decision making on the GI liability of compounds in development.

**Methods:**

Fifteen compounds associated with or without clinical GADRs were used to assess the ability of an *in vitro* colonic motility bioassay to predict motility-related GADRs. Compounds were examined in a blinded fashion for their effects on mouse colonic peristaltic motor complexes *in vitro*. For each compound concentration-response relationships were determined and the results compared to clinical data. Compounds were also assessed using GI transit measurements obtained using an *in vivo* rat charcoal meal model.

**Key Results:**

Within a clinically relevant dosing range, the *in vitro* assay identified five true and three false positives, four true and three false negatives, which gave a predictive capacity of 60%. The *in vivo* assay detected four true and four false positives, four false and three true negatives, giving rise to a predictive capacity for this model of 47%.

**Conclusions & Inferences:**

Overall these results imply that both assays are poor predictors of GADRs. Further analysis would benefit from a larger compound set, but the data show a clear need for improved models for use in safety pharmacology assessment of GI motility.

**Key Messages**

- Gastrointestinal adverse drug reactions (GADRs) such as diarrhea and constipation pose a genuine clinical issue for drug discovery programs, prompting the need to design simple and reliable biomarkers of gastrointestinal (GI) function.
- The main findings from this study were that a novel *in-vitro* bioassay and an established *in-vivo* charcoal meal model were poor predictors of GADRs when both models were assessed using a series of 15 compounds associated with/without clinical GADRs.
- Our observations suggest that improved models are required for safety pharmacology assessments of motility-related GADRs.

## Introduction

Gastrointestinal adverse drug reactions (GADRs) represent around 67% of adverse drug reactions (ADRs) described on drug labels^1^ and account for 23% of adverse events encountered in phase I studies.^2^ Motility-related GADRs such as diarrhea and constipation are common side-effect profiles of marketed drugs,^3^–^6^ and account for a high proportion of ADRs encountered during the development of new chemical entities.^7^ Gastrointestinal adverse drug reactions can be dose limiting preclinically and/or clinically, affect patient compliance, or even lead to compound discontinuation.^8^ Consequently, GADRs pose a genuine clinical issue and are contributing to the escalating costs of developing new drugs.^9^,^10^ Accurate and simple biomarkers of gastrointestinal (GI) function are therefore required to detect potential GADRs earlier in the drug discovery pathway and so maximize the opportunity to ‘design out’ this undesirable characteristic, or to deselect compounds from on-going development. Ultimately, this should deliver new drugs that have improved patient compliance and commercial attractiveness.

Presently, the GI tract is classified as a supplementary organ system by the International Congress on Harmonisation (ICH) section 7A guidelines, the regulatory requirement that must be fulfilled before testing novel pharmaceuticals in humans. As a result, pharmaceutical companies do not routinely assess the effect of new chemical entities on the GI tract before clinical development, and if they do, they are often not considered as decision making studies. However, the high incidence of GADRs and their impact upon patient safety and compliance, in addition to payer requirements and commercial attractiveness, is now challenging this approach.

A number of *in vivo* and *in vitro* models are available to investigate GI function.^11^–^14^ In addition, newer screening approaches have been proposed based on zebrafish^15^,^16^ and more established rodent-based models.^17^ Recently, we described a novel *in vitro* bioassay for use as a model of motility-related GADRs (diarrhea/constipation). This bioassay employs segments of mouse colon which generate robust and stereotypic patterns of peristaltic motor complex activity when mounted in an organ bath.^18^,^19^ Using a series of compounds with well-validated GI motility-related effects, we demonstrated that this model could potentially aid GI safety pharmacology assessment. However, before routine use in a safety pharmacology screening cascade, we wanted to investigate its utility as an early hazard warning tool.

Therefore, the aim of this investigation was twofold: (a) to determine the concordance, at clinically relevant concentrations, between the *in vitro* mouse bioassay and phase I clinical outcome of compounds where the molecular target was not necessarily associated with GI motility and (b) to investigate whether the *in vitro* mouse bioassay performed any differently to the charcoal meal test, the current method of choice for determining the effects of compounds on GI transit *in vivo* in rodents.^20^

## Methods

### Clinical studies

Fifteen compounds were used in this study, of which 13 were proprietary AstraZeneca compounds, while the remaining two were in clinical use (Table 1). Clinical data were gathered on these compounds including GI symptoms, incidence, and plasma exposure (Table 1).

**Table 1:** Clinical data including type of GADRs, % of subjects affected, and exposure range

Drug	Clinical finding	% subjects affected	Minimum and maximum free exposure range (*μ*M)
B	None	0	0.0024–0.6597
C	Constipation	17	0.0010–0.0029
D	None	0	0.0050–0.0352
E	None	0	0.0060–2.6331
F	Diarrhea	67	0.00007–0.6051
G*	Diarrhea	53	6–12
H	Diarrhea	17	0.0006–0.0710
I*	Loose stools	25	0.7–18.9
J	None	0	0.000008–0.0008
M	Diarrhea	17	0.0017–0.0506
N*	None	0	0.00014–0.0373
R	None	0	0.000009–0.0837
S	Nausea	13	0.000125––0.001
T	Diarrhea	67	0.00935–0.1837
Z	Loose watery stools	100	0.00027–2

* Total plasma exposure. % affected is at the highest dose level. Free exposure range refers to the mean exposure at the lowest and highest dose tested clinically.

### Ethical approval for the assay development

All experimental procedures were conducted with local ethical committee approval and in accordance with the UK Animals (Scientific Procedures) Act, 1986 (National Archives, UK Animals, Scientific Procedures Act, 1986) under the authority of valid Home Office project licences.

### *In vitro* colonic motility bioassay: experimental protocol

These experiments were performed at the University of Sheffield. Ninety-nine adult male mice (C57Bl/6J, bred in-house at the University of Sheffield, Sheffield, UK), 6–8 weeks old and weighing ∼24 g were used. Animals were housed under controlled ambient temperature (21 ± 2 °C) and light–dark cycle (12 : 12 h) and were allowed free access to food and water.

**Figure 1:**
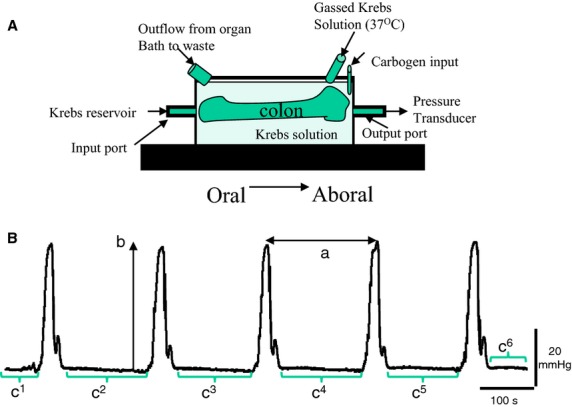
Representative trace of basal murine colonic peristaltic motor complexes (CPMCs). (A) Schematic diagram of the *in vitro* organ bath experimental apparatus. (B) The parameters used to evaluate changes in motility are illustrated; *a*, interval between successive CPMCs; *b*, amplitude of individual CPMC; *c*^*1*^*+c*^*2*^*+etc*., time in quiescence (TIQ). The AUC represents the total area under the curve for the 15-min analysis period, while the frequency represents the number of CPMC measured during this time period.

The methodology has been described in detail previously.^19^ Briefly, animals were euthanized by cervical dislocation. A midline laparotomy was immediately performed and the exposed abdomen bathed in Krebs solution (in mM: 119 NaCl, 4.7 KCl, 1 NaH_2_PO_4_, 1.2 MgSO_4_, 25 NaHCO_3_, 2.5 CaCl_2_, 11 glucose) gassed with carbogen (95% O_2_, 5% CO_2_). The entire colon was removed and immediately placed into a beaker of Krebs solution. Using a syringe, the lumen was then cleared off any contents by gentle flushing with Krebs solution. An adapted standard organ bath procedure was used. Segments of colon (6 cm length) were placed into the organ bath (20 mL volume) perfused continuously (10 mL/min) with 37 °C Krebs solution, gassed with carbogen, and equilibrated to pH 7.4. The oral and aboral ends of the colon segment were securely attached to an input and output port of the organ bath, respectively (Fig.1A). The input port was connected to a reservoir and syringe setup which allowed the controlled perfusion of Krebs solution through the lumen of the colonic segment, while the output port was attached in series to a pressure transducer (BD DTXPlus™, Oxford, UK). Motor activity was initiated in the colon segments by an infusion of Krebs from the syringe into the lumen of the colon until an intraluminal pressure (IP) of ∼5 mmHg had been reached. Under these conditions, regular aborally propagating waves of contraction developed spontaneously and persisted over time. These contractile waves were recorded as changes in IP (Fig.1B) and were termed colonic peristaltic motor complexes (CPMCs), similar to the normal peristaltic activity of the colon. After an equilibration period of 60 min, CPMC activity had reached a consistent pattern, in terms of their amplitude and frequency, and experimental procedures were then started. A very small number of tissues (∼2% or less) failed to show robust CPMC-like activity and these were discarded from use before any experimental procedures took place.

### *In vitro* bioassay: experimental procedure

Test compounds were bath applied using a cumulative concentration-response protocol consisting of four successive 15-min perfusion periods, starting with the vehicle and three incremental concentrations of a drug, followed by a washout period to assess the reversibility of effects. Compounds were tested at 0.3, 3, and 30 *μ*M and their effects upon CPMC parameters were noted. If a compound gave rise to significant excitatory or inhibitory effects at the lowest concentration tested (0.3 *μ*M), the concentration range was reduced and experiments repeated using a cumulative dosing procedure at the lower concentration range. This strategy was repeated until a cumulative concentration-response effect for each drug had been obtained. The concentration-range details for these experiments are provided in Table 2 and Table S1. A series of time-matched controls were also performed using serial dilutions of DMSO in Krebs solution and the details of these are also included in Table S1.

**Table 2:** Summary data for test compounds examined in the *in vitro* assay with comparison to clinical data

Drug	Drug concentration tested in assay (*μ*M)	Clinical finding	Predictive outcome maximum dose	Predictive outcome therapeutic dose
B (*N* = 8)	0.3–30	NE	FP	FP
C (*N* = 6)	0.003–0.3	C	TP	TP
D (*N* = 6)	0.3–30	NE	FP	TN
E (*N* = 6)	0.3–30	NE	FP	FP
F (*N* = 6)	0.3–30	D	TP	TP
G (*N* = 7)	0.3–30	D	FN	FN
H (*N* = 7)	0.3–30	D	TP	TP
I (*N* = 7)	0.3–30	D	FN	FN
J (*N* = 6)	0.03–3	NE	FP	FP
M (*N* = 6)	0.3–30	D	TP	FN
N (*N* = 6)	0.3–30	NE	FP	TN
R (*N* = 6)	0.3–30	NE	TN	TN
S (*N* = 4)	0.3–30	NE	FP*	TN*
T (*N* = 6)	0.3–30	D	TP	TP
Z (*N* = 6)	0.3–30	D	TP	TP

* Minimum testing concentration was ×300-fold plasma exposure. C, constipation; D, diarrhea; TN, true negative; TP, true positive; FP, false positive; FN, false negative; NC, no change; NE, no effect.

### CPMC activity quantification

Colonic peristaltic motor complex activity was quantified using a set of five parameters calculated for each 15-min experimental phase of the cumulative concentration experiments. These were as follows: CPMC frequency (number of CPMCs/15 min), the average time interval between successive CPMCs (TI, s), the time in quiescence (TIQ, s), which is a measurement of the total time that the tissue is at baseline activity during the 15-min period of drug or vehicle perfusion, the average CPMC amplitude (mmHg), and the area under curve (AUC, mmHg/s) (Fig.1B). The AUC and amplitude reflected the mechanical activity of the tissue, whereas the TIQ reflected the overall time with motor activity.

### Data analysis

Changes in IP generated by CPMC activity were amplified (Digitimer, NL108, Welwyn Garden City, UK) and subsequently acquired to a computer through a CED 1401 interface and Spike2 software (Cambridge Electronic Design, Cambridge, UK). Intraluminal pressure was sampled at 100 Hz. Analysis was carried out off-line using the software applications contained in the Spike2 software package. AUC was calculated using an in-built script in the Spike2 software. Raw data are expressed as means ± SEM and compared by one-way anova for repeated measures followed by Dunnett's test as appropriate. *p* < 0.05 was taken as significant. Data for TIQ and amplitude are also expressed as percentage changes relative to their corresponding vehicles (relative changes), and were compared to relative changes in TIQ and amplitude calculated from time-matched vehicle control experiments. Compounds which elicited relative changes in either CPMC amplitude or TIQ that were >2 SD from the mean time-matched control values were taken as positive responding compounds. Relative changes above 2 SD were used as a decision criterion due to the small range of TIQ and amplitude values observed in the time-matched controls and compound experiments.

### Drugs

All compounds were obtained from AstraZeneca in coded vials and were tested in a blinded fashion (the number of experiments conducted is indicated in parentheses in Tables 2 and 3). Stock solutions of 1 or 10 mM were prepared by adding an appropriate volume of either H_2_O or DMSO and were diluted to test concentrations in Krebs solution. The maximum concentration of DMSO used was 0.1%. Drugs were applied to the serosal surface and while the bath concentrations of each compound were not measured during the experiments, they were assumed to reach a steady-state concentration equal to that of the perfusate.

**Table 3:** Summary data for test compounds examined in the charcoal meal assay with comparison to clinical data

Drug	Drug concentration measured in model (*μ*M)	Charcoal meal effect*	Clinical finding	Predictive outcome
B (*N* = 10)	0.08–22	Decrease	NE	FP
C (*N* = 8)	0.06–0.58	NC	C	FN
D (*N* = 8)	0.196	NC	NE	TN
E (*N* = 8)	0.28–5.38	Decrease	NE	FP
F (*N* = 8)	0.072–0.183	Decrease	D	TP
G (*N* = 8)	1.67–468	Decrease	D	TP
H (*N* = 8)	0–0.0014	Decrease	D	TP
I (*N* = 8)	11.3–189	Decrease	D	TP
J (*N* = 8)	0.012–0.034	Decrease	NE	FP
M (*N* = 8)	69	Decrease†	D	FN
N (*N* = 8)	0.44	NC	NE	TN
R (*N* = 8)	0.06–10	NC	NE	TN
S (*N* = 8)	0–0.0000273	Increase	NE	FP
T (*N* = 10)	15–344	NC	D	FN
Z (*N* = 10)	0–23	NC	D	FN

* Data refer to the assay effects recorded at testing concentrations of <50-fold the equivalent plasma exposure.

† Only one dose tested at an exposure of 69 *μ*M (>1000-fold the equivalent plasma exposure). Abbreviations are the same as for Table 2.

### Charcoal meal *in vivo* test: experimental protocol

These studies were performed at AstraZeneca R&D Alderley Park, UK. Male Han Wistar rats weighing between 230 and 270 g (AstraZeneca Breeding Unit, Alderley Park, UK or Harlan Laboratories UK Ltd, Bicester, Oxon, UK) were housed in groups of 4 with free access to food and water. Animals were housed under ambient temperature (19–23 °C), relative humidity (40–70%), and a 12-h light/dark cycle. At the start of the 6-h fasting period, animals were placed in cages with a grid floor. The measurements of GI function were performed in the afternoon (∼14:00 h) and the time of food removal was altered to fit the designated fasting period accordingly.

### Charcoal meal *in vivo* test: drugs and experimental procedure

The methodology for the *in vivo* testing has been described previously.^17^ All compounds were administered orally (dose levels are indicated in Table S2). At the approximate *T*_max_ of each individual compound, animals were administered a charcoal meal (activated charcoal; Sigma, Poole, Dorset, UK) formulated as a suspension in 2% w/v carboxymethylcellulose (10 : 90). Fifteen to 20 min later, they were killed by an overdose with halothane followed by exsanguination. Following midline laparotomy, the esophagogastric (cardia), gastroduodenal (pyloric sphincter), and ileo-cecal junctions were ligated, and the stomach and small intestine carefully removed from the esophagogastric to the ileo-cecal junction. Each stomach, including contents, was weighed and then cut open and rinsed with physiological saline. Excess moisture was removed by gentle sponging with laboratory tissue and the empty stomach weighed. Index of gastric emptying (g) = full stomach weight (g) − empty stomach weight (g). The entire small intestine was gently stretched out and the total length measured. The distance traveled by the charcoal meal from the pyloric sphincter to the ileo-cecal junction was measured.

### Intestinal transit quantification and data analysis

Intestinal transit was defined as the position of the leading edge of the charcoal expressed as a percentage of the total length of the small intestine, calculated as follows: Intestinal transit distance (%) = [distance traveled by charcoal (cm)/length of small intestine (cm)] × 100.

The results from the charcoal meal were expressed as medians. A non-parametric test (Wilcoxon Mann–Whitney test) was used to compare the compound-treated groups with the vehicle-treated group, as the distribution of the data was unknown. *p* < 0.05 was taken as significant. Where possible, free plasma concentrations were calculated, but for three compounds (G, I, and N), the plasma protein binding was not known (but thought to be negligible) and therefore, the plasma exposure data are shown as a total value.

### Assay quantification

The compounds tested in the two assays were assigned to the following categories by comparing their experimental effects to their clinical effects. To avoid any potential bias, the clinical effects of these compounds were withheld from the investigators until after the CPMC and intestinal transit quantification stages were completed. The categories were as follows: true positives (TP) were compounds which caused GADRs and elicited relative changes to either CPMC amplitude or TIQ that were >2 SD from their mean control values, or caused significant changes to intestinal transit. False negatives (FN) were compounds which caused GADRs and elicited relative changes in amplitude or TIQ that were within 2 SD of their mean control values, or had no significant effect on intestinal transit. True negative (TN) compounds did not cause GADRs or elicit changes in either relative CPMC activity or intestinal transit activity that indicated positive compound effects. False positives (FP) are compounds which possessed no clinically detected GADR activity, but elicited changes in relative CPMC activity or intestinal transit that were indicative of positive compound effects.

The predictive capacity (defined as the proportion of correctly identified results) was calculated as follows: Predictive capacity = (TP+TN)/total number of compounds tested.^21^,^22^

## Results

### Clinical effects of the compounds

Clinical data were gathered on 15 compounds, including 13 proprietary AstraZeneca compounds, and two compounds currently used in clinical settings. Eight of the compounds caused diarrhea or constipation affecting between 17% and 100% of the patients, while seven had no reported GADRs such as diarrhea or constipation although one, rimonabant (Compound S), was reported to cause nausea (Table 1).

### CPMC activity

Baseline data on CPMC activity (collected over a 15-min period from a total of 99 experiments) are summarized as follows: frequency (the number of CPMCs/900 s), 5.6 ± 0.15; TI (the time between individual CPMCs), 161.4 ± 4 s; TIQ, 661 ± 6 s; amplitude (CPMC amplitude), 49 ± 1.5 mmHg; AUC (total activity during 900-s period, AUC), 6410 ± 307 mmHg/s.

### Compound effects upon CPMC activity

**Figure 2:**
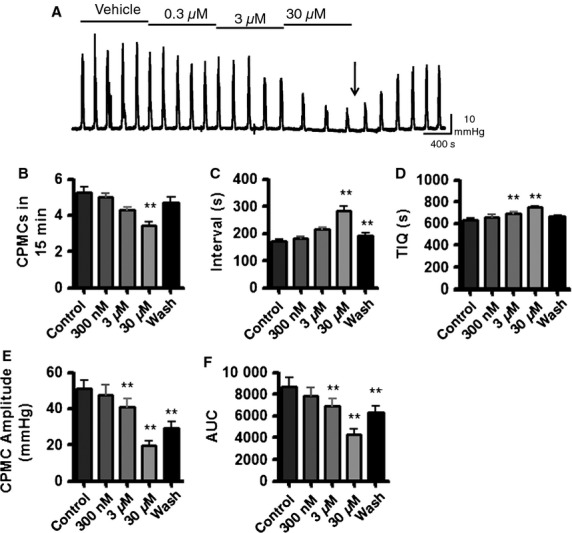
Effects of Compound H upon colonic peristaltic motor complex (CPMC) activity. (A) Representative recording showing the effects of cumulative additions of Compound H upon CPMC activity. Downward arrow represents start of washout period. CPMC activity is decreased in a concentration-dependent fashion. (B–F) Illustrate the concentration-dependent effects of Compound H (*n* = 7) on the frequency (B); the time between consecutive CPMCs (C); time in quiescence (TIQ; D); the amplitude of CPMCs (E); and the AUC (F). Data are expressed as mean ± SEM; *p < 0.05; **p < 0.01 *vs* control by repeated measures one-way anova.

**Figure 3:**
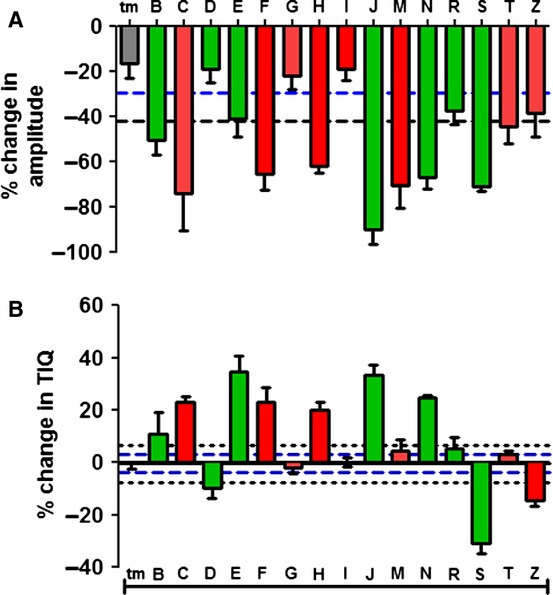
(A) Colonic peristaltic motor complex (CPMC) amplitude for test compounds plotted as relative changes in amplitude at their maximum dose compared to vehicle. Compounds are plotted alongside appropriate time-matched vehicle control data (*N* = 4). (B) Similar analysis for time in quiescence (TIQ) in which relative TIQ values for the test compounds are plotted alongside appropriate time-matched control data. Red bars indicate compounds associated with GADRs. Green bars represent compounds with no GADRs reported. Black dashed line represents 2 SD difference from the mean value. Blue dashed line represents 1 SD difference from the mean control value.

The 15 compounds were tested in a blinded fashion for their effects upon CPMC activity. Two compounds (C and J) elicited effects at the lowest dose tested and were retested using a lower dosing strategy. The remaining compounds were tested using the standard dosing regimen. The raw data from these experiments are summarized in Table S1 and an illustrative example is shown in Fig.2. The actions of the test compounds upon CPMC amplitude and TIQ were also plotted as percentage changes relative to the vehicle response (relative change) and were compared to appropriate time-matched controls (Fig.3).

### Effect of drugs on relative changes in CPMC parameters

At the highest drug dose tested, nine compounds elicited relative amplitude changes greater than ±2 SD of the mean amplitude change for time-matched controls (Fig.3A). A further three compounds elicited relative amplitude changes greater than ±1 SD of the mean control amplitude change, while three compounds had effects indistinguishable from control amplitude values (Fig.3A). A similar analysis of the TIQ data showed that 10 compounds gave rise to relative changes in TIQ > ±2 SD of the mean TIQ change for time-matched controls (Fig.3B). Two compounds elicited effects that were greater than ±1 SD of the mean control amplitude change, while three compounds had effects that were indistinguishable from controls (Fig.3B).

### Effects of test compounds on relative changes at therapeutic concentrations

Relative changes in TIQ and amplitude were also assessed within clinically relevant concentrations (defined as concentrations within 50-fold of the maximum plasma exposure recorded in the clinical trials). When relative amplitude and TIQ changes were assessed at the highest clinically relevant dose tested, three compounds (B, F, and T) elicited relative amplitude changes and seven compounds (B, C, E, F, H, J, and Z) elicited relative TIQ changes that were greater than ±2 SD from their mean time-matched control changes. Several of the remaining compounds (D, M, and S) elicited relative changes in one of the parameters that was greater than ±1 SD amplitude of the mean time-matched control data, while compounds G, I, N, and R gave rise to responses that were indistinguishable from time match control data.

### CPMC assay translation

The compounds' effects on relative changes to either amplitude or TIQ were compared to their clinical actions and the predictive capacity of the assay calculated. The assay was found to have a predictive capacity of 47% at the maximum drug concentration tested and where the effect of drug exceeded 2 SD of the control. This reflected the identification of 6 TPs, 2 FNs, 1 TN, and 6 FPs (Table 2). However, at the clinically relevant concentrations, the assay identified 5 TPs, 3 FNs, 4 TNs, and 3 FPs, which gave a predictive capacity of 60% (Table 2). A contingency analysis of these results revealed no significant differences between the two data sets (*p* > 0.05).

### Effects of compounds on the *in vivo* charcoal meal model

Based on an internal AstraZeneca database, the baseline median small intestinal transit time of rats (*n* = 287) fasted for 6 h, and treated with a vehicle, prior to a charcoal meal was 53.3 ± 12.2%.^23^ Of the 15 compounds tested in the *in vivo* charcoal meal study, eight compounds (B, E, F, G, H, I, J, and S) caused significant changes in intestinal transit at clinically relevant concentrations (defined as concentrations within 50-fold of the maximum plasma exposure recorded in the clinical trials) and these data are recorded in Table 3 and Table S2. Only compound S stimulated intestinal transit, while the remaining compounds were associated with inhibition (Table 3 and Table S2). Five compounds (D, N, R, T, and Z) had no effect upon intestinal transit, while two compounds (C and M) inhibited intestinal transit, but at doses >50-fold the maximum plasma exposure (Table 3 and Table S2). Several of these compounds were examined for their effects upon gastric emptying and we found that all the compounds tested in this group altered gastric emptying (Table S2).

### Charcoal meal assay translation

An analysis was performed for the charcoal meal test in which 4 TPs (Compounds F, G, H, and I), 4 FNs (Compounds C, M, T, and Z), 3 TNs (Compounds D, N, and R), and 4 FPs (Compounds B, E, J, and S) were identified, giving rise to a predictive capacity for this model of 47% (Fig.3, Table 3).

## Discussion

This study set out to determine the translational potential of a mouse *in vitro* bioassay and evaluate its potential as an early screen to detect GI liability in novel compounds. We focused on analyzing relative changes in either amplitude or TIQ to assess the predictive capacity of the model during the course of this study. Measuring amplitude gave an indication of the effects of the compounds upon CPMC contractility, while TIQ assessed the pattern of CPMC activity. We focused upon using TIQ as a metric as our previous study showed that TIQ was a robust and stable marker of CPMC frequency.^19^ In our initial screen in which absolute changes in CPMC activity were determined, it was revealed that all the compounds significantly affected amplitude at the highest drug dose tested. However, we were mindful of over interpreting these data as time-matched control experiments also revealed a small but significant time-dependent change in amplitude. This led to concerns that these time-dependent amplitude changes were confounding the drug-induced effects on this parameter, and so by plotting relative changes in CPMC amplitude and comparing these to equivalent time-matched controls, we were able to control for these confounding factors.

Initially, the predictivity of the assay was determined using relative changes observed at the highest dose tested. This may seem counter intuitive as we knew the plasma exposures measured in the phase I studies, but this assay was designed as an early safety screen in which the therapeutic exposures of test compounds would not be known. Consequently, we wished to observe whether the assay would be effective at the upper limit of testing conditions. Under these conditions, the assay was found to have a predictive capacity of 47%, while at clinically relevant exposures, the predictive capacity was 60%. The difference between these two assessments reflected an increased number of FPs detected at the high testing concentrations, although no significance was found between the two methods of analysis.

Overall the predictive capacity of the assay was considered ‘insufficient’ according to the European Centre for the Validation of Alternative Methods criteria for validating *in vitro* toxicology screening methods^21^ and most likely reflect the fact that the low sensitivity and specificity of the assay (the relatively high proportion of FNs and FPs detected) impacted negatively upon its validity as an early screening model.

*In vitro* assays have several advantages for screening such as relatively low costs and a capacity for medium to high throughput testing. However, their predictive capacity may be limited by their incomplete physiological nature. For example, the isolated colon lacks central nervous system neuronal influences, in addition to systemic hormonal or metabolic influences and as such the *in vitro* assay measures direct actions of compounds upon colonic motility. Therefore, compounds which cause changes in motility through centrally mediated pathways would have no effect in this model. This is illustrated in the results of Compound G which was the drug metformin and was one of only two of the compounds whose identity was revealed in this study. Metformin is a hypoglycemic agent used to treat type 2 diabetes.^24^,^25^ Its clinical use is associated with dose dependent GI disturbances including diarrhea,^26^ and the mechanisms contributing to this GI dysfunction are thought to involve metabolic and centrally mediated effects.^27^

We found that metformin had no significant effect upon CPMC activity, but caused diarrhea in 53% of the subjects tested. Consequently, Compound G was incorrectly identified as a FN. These findings highlight an important limitation inherent in all *in vitro* assays in that compounds which produce their effects via centrally mediated mechanisms would have no effect in these *in vitro* models, but they could be detected using appropriate *in vivo* techniques. Interestingly, when this same compound was tested in the charcoal meal model, the correct phenotype was identified demonstrating a need to employ complementary screening strategies throughout the drug discovery process.

Despite these limitations, we did find that the *in vitro* assay detected ‘high-incidence’ compounds better than ‘low incidence’ compounds. Four of the eight compounds associated with causing GADRs affected a high percentage of the patients (>50% affected), while the remaining compounds were associated with lower incidences (<25% of the cohort affected). The assay correctly identified three of these ‘high-incidence’ compounds, while in the ‘low incidence’ category, only two compounds were correctly identified. It is likely that other factors contribute toward these low incidences of GADRs including stress, diet, or environmental factors and these cannot be modeled *in vitro*. Two of the compounds associated with high incidence of GADRs (Compounds T and Z) were withdrawn from development due to their side effects and both these compounds were flagged by the assay as having potential GI liabilities. Therefore, despite the limitations associated with this assay, it appears that this model does have the capacity to act as a hazard flag in certain situations, and as such could form a useful addition to a battery of GI-orientated safety screens.

This study showed that only Compound Z increased CPMC activity, in accordance with its diarrheal phenotype, while for the remaining compounds, CPMC activity was decreased, even if diarrhea was the clinical response to exposure of these drugs. While we would have expected compounds causing diarrhea to be associated with increased CPMC activity, so to drive increased propulsion, contractions can both propel and impede flow. As such, we sought only to determine if a quantifiable change in CPMC activity could be detected, and the extent to which this was correlated with GADRs irrespective of whether this was constipation or diarrhea.

The concentration relationship between the clinical settings and the assay is important in determining safety margins. We used a 50-fold margin between the minimum assay drug concentration required to elicit significant responses and the maximum plasma concentration achieved in patients as a therapeutic window to ensure relevant safety margins and also limit the risk of obtaining FPs. This range corresponds to limits set in other, notably cardiovascular, safety models that suggest a 30-fold margin between the highest free plasma concentration of a drug and safety assay responses were adequate for this purpose.^1^,^23^,^28^ Interestingly, Compound I was tested at a maximum concentration that was only twofold greater than the maximum therapeutic dose used, and it is conceivable that this dose was too low to cause any significant effects upon CPMC activity which may explain the FN phenotype associated with this compound.

Seven of the compounds tested in this study had no GADRs associated with them, and using our therapeutic dosing limits the assay correctly identified four of these compounds as TNs. It is important to note that while these compounds did alter CPMC activity, these effects were seen at dosing margins of above 100-fold, and in some cases greater than 1000-fold. However, three compounds (B, E, and J) elicited effects upon CPMC activity at clinically relevant doses and were designated as FPs. False positives are a major issue for a safety screen as it can lead to the discontinuation of safe compounds which could potentially be life-saving medicines. Both models identified compounds B, E, and J as FPs suggesting that physiological pathways present in rodents, but absent in humans contribute to this difference. In the absence of any pharmacological knowledge of these compounds, we cannot speculate on why these results occurred, but they reflect the importance of designing safety screening systems to counteract possible species-related differences in the drug target biology.

The results from this study suggested that the *in vitro* assay lacks the overall predictivity to be utilized as a screening model. We were also able to test the clinical compounds using the charcoal meal test,^11^ the current gold standard in safety assessment testing, and surprisingly we found that this assay was also a poor predictor of GADRs in the clinic. Direct comparisons of the two models were avoided as they were performed in different species, but the charcoal meal test likely possesses some inherent experimental advantages over an *in vitro* technique such as being capable of detecting compounds acting via central processes. However, the *in vitro* assay also offers some experimental benefits. For example, the *in vitro* assay allows a more thorough physiological assessment of drug-induced changes in motility (amplitude *vs* pattern of activity as opposed to overall transit) than the *in vivo* method, and this could provide valuable information to the mechanisms through which compounds are affecting motility. This was clearly demonstrated in our previous study in which compounds could be discriminated by the *in vitro* model as myogenic or neurogenic activators.^19^

### Conclusion

Fifteen marketed drugs or drugs intended for market, which are associated with or without GADRs, were tested in a blinded manner in an *in vitro* bioassay which we proposed as a biomarker for predicting the GADR liability of new chemical entities. The *in vitro* assay performed similarly to an *in vivo* model in detecting compounds associated with GADRs in phase 1 trials, and while the *in vitro* assay shows some promise in detecting high risk compounds, currently both techniques are unacceptable in terms of their overall accuracy as stand-alone bioassays to detect GADRs. Consequently, more efforts are needed to develop suitable assays, or possibly a combination of assays, to detect the GI motility liability of novel compounds in development.

## Abbreviations

ADR: adverse drug reaction
AUC: area under curve
CNS: central nervous system
CPMCs: colonic peristaltic motor complexes
FN: false negative
FP: false positive
GADRs: gastrointestinal adverse drug reactions
GI: gastrointestinal
IP: intraluminal pressure
TIQ: time in quiescence
TN: true negative
TP: true positive

## SUPPORTING INFORMATION

**Table S1.** Functional characteristics for all compounds tested in the mouse *in vitro* assay. **Table S2.** Intestinal transit data for all compounds tested in the rat charcoal meal study.
